# Visual assessment of movement quality: a study on intra- and interrater reliability of a multi-segmental single leg squat test

**DOI:** 10.1186/s13102-021-00289-x

**Published:** 2021-06-08

**Authors:** John Ressman, Wilhelmus Johannes Andreas Grooten, Eva Rasmussen-Barr

**Affiliations:** 1 Department of Neurobiology, Karolinska Institutet, Care Sciences and Society, Division of Physiotherapy, Alfred Nobels Allé 23, 141 83 Huddinge, Sweden; 2 Women’s Health and Allied Health Professionals’ Theme, Karolinska University Hospital, Solna, Stockholm 171 76 Sweden

**Keywords:** Single leg squat, Visual assessment, Movement quality, Reliability, Functional tests, Kappa, Reproducibility

## Abstract

**Background:**

The Single Leg Squat test (SLS) is a common tool used in clinical examination to set and evaluate rehabilitation goals, but there is not one established SLS test used in the clinic. Based on previous scientific findings on the reliability of the SLS test and with a methodological rigorous setup, the aim of the present study was to investigate the intra- and interrater reliability of a standardised multi-segmental SLS test.

**Methods:**

We performed a study of measurement properties to investigate the intra- and interrater reliability of a standardised multi-segmental SLS test including the assessment of the foot, knee, pelvis, and trunk. Novice and experienced physiotherapists rated 65 video recorded SLS tests from 34 test persons. We followed the Quality Appraisal for Reliability Studies checklist.

**Results:**

Regardless of the raters experience, the interrater reliability varied between “moderate” for the knee variable (ĸ = 0.41, 95% CI 0.10–0.72) and “almost perfect” for the foot (ĸ = 1.00, 95% CI 1.00–1.00). The intrarater reliability varied between “slight” (pelvic variable; ĸ = 0.17, 95% CI -0.22-0.55) to “almost perfect” (foot variable; ĸ = 1.00, 95% CI 1.00–1.00; trunk variable; ĸ = 0.82, 95% CI 0.66–0.97). A generalised kappa coefficient including the values from all raters and segments reached “moderate” interrater reliability (ĸ = 0.52, 95% CI 0.43–0.61), the corresponding value for the intrarater reliability reached “almost perfect” (ĸ = 0.82, 95% CI 0.77–0.86).

**Conclusions:**

The present study shows a “moderate” interrater reliability and an “almost perfect” intrarater reliability for the variable all segments regardless of the raters experience. Thus, we conclude that the proposed standardised multi-segmental SLS test is reliable enough to be used in an active population.

**Supplementary Information:**

The online version contains supplementary material available at 10.1186/s13102-021-00289-x.

## Background

In the clinical setting, visual assessment of movement quality is one of the most commonly used methods to examine patients, and to evaluate and target rehabilitation goals. The term movement quality is often used in relation to the visual assessment of asymmetries, compensatory movements, impairments, and efficiency during a functional movement [1, 2]. Movement quality is described as an independent attribute, and unlike quantitative measures such as power and strength, movement quality aims to capture other important aspects of the movement [1, 3, 4]. This is recommended for example in the rehabilitation of anterior cruciate ligament injuries where the assessment of quantitative as well as qualitative aspects are recommended in the decision of a safe return to play [5]. In addition, observation of the alignment of body segments and the maintenance of a correct posture is often included in the assessment of movement quality [4, 6, 7], and malalignments of the lower extremity segments are often seen in knee injuries and other overuse injuries [8–13].

The Single Leg Squat test (SLS) is a functional movement test widely used in clinical settings to visually assess movement quality of the lower extremity and is proposed to have biomechanical and neuromuscular similarities to a wide range of athletic movements as it simulates common athletic positions such as cutting, jumping, and landing [14, 15]. It is also commonly included in various screening and test batteries used in sports medicine [16–19]. The SLS test has been named, described, performed, and assessed in many different ways, meaning that there is not one established SLS test [20]. Reported performance differs in many aspects of the test, such as depth of the squat, position of the arms, support and the position of the non-weight bearing leg (in front, behind or below the trunk) [18, 21–26]. In addition to the SLS test, the Forward Step Down (FSD) and Lateral Step Down (LSD), are tests performed on a 15–25 cm high box but otherwise performed and assessed in the same manner as the SLS test [23, 27]. Although the movement pattern during the descendent phase of a SLS, FSD or LSD are the same [28, 29], different kinematic and kinetic have been reported between the SLS tests [28], the SLS test and FSD [29] and in addition between men and women [30, 31]. One important aspect is the position of the non-weight bearing leg where the behind position seems to have the most kinematic differences from the front or below position [28].

The SLS test has been reported to be reliable and valid in clinical and research settings for an asymptomatic healthy population when assessing the knee in relation to the foot [20, 21, 32, 33]. In addition, a multi-segmental approach was recently proposed to be feasible and reliable, preferably with a two- or three-point rating scale [20]. The reliability of the SLS test has previously been explored by either rating video recordings of the test or by rating the performance live.

A reference method for measuring movements are 3-dimensional (3D) analysis systems or 2-dimensional (2D) techniques, however not accessible for all clinicians, and is in addition time consuming, impractical, and not applicable in a larger population [34]. Thus, it is important to further develop movement quality tests used in the clinic regarding their measurement properties.

It would be desirable to evolve a less complex and well-defined SLS test, which is easy to use regardless of the examiner’s education or clinical experience. The interpretation of the SLS test should in addition comprise a distinct protocol on how to rate the movement. We propose a SLS test, taking the visual assessment of the kinetic chain from the foot to the trunk into consideration; a multi-segmental approach which might give the clinician further information in the clinical assessment and targeted rehabilitation [20, 33]. In the proposed test, we have included an item considering the position of the foot, in contrast to most other SLS tests, as we believe that the foot position affects the alignment of the kinetic chain. The proposed SLS test is based on the findings from two previous meta-analyses on the validity and reliability of visually assessed ratings on the lower extremity [32, 33], and in addition a recent meta-analysis on the intra- and interrater reliability of the SLS test [20]. Recent studies on the reliability of the SLS test have reported poor methodological quality, thus further studies with more robust methodological standardisation are warranted [20]. Based on previous scientific findings on the reliability of the SLS test and a robust methodological standardisation, the aim of the present study was to investigate the intra- and interrater reliability of a standardised multi-segmental SLS test.

## Methods

### Study design

This study investigated the intra- and interrater reliability of video-recorded SLS tests and followed the Quality Appraisal for Reliability Studies checklist (QAREL) [35] which can be found in Additional file 1.

### Subjects

Thirty-seven healthy persons (27 women, 10 men) aged 34 (±12) years were recruited via verbal announcements and informational posters at the Karolinska Institutet in Stockholm. Inclusion criteria were men and women, aged 18 to 65. Exclusion criteria were an ongoing musculoskeletal injury in the lower extremity, a history of serious knee disorder (ligament- or meniscal rupture and knee replacement), a neurological disease, or a visual deficiency that could not be corrected with eyeglasses. A written informed consent to agree to participate in the study was obtained for all individual subjects. The study was approved by the Regional Ethical Review Board in Stockholm: Ethical approval Dnr: 2016/595–31 with amendment Dnr 2017/318–32 and the Karolinska Institute to which the ethical approval belongs.

### Data collection

Before performing the SLS test, all test persons filled in a questionnaire concerning demographic and background data. The tests were performed in the movement laboratory of the Karolinska Institutet during 21 March and 11 May 2017, and administrated by two of the authors (JR and WG). The SLS tests was recorded in the frontal and the sagittal plane with two orthogonally placed digital video cameras (Axis Communications 210A) at three metres’ distance. The cameras were placed so that the whole body was visible, with a brown even background.

#### The SLS test

The test persons were first verbally instructed on how to perform the SLS test by one of the test leaders (JR) and was then allowed to practice the test for three times. When performing the test, the test person followed a pre-recorded video clip with precise verbal instructions on how to perform the test (Additional file 2). All participants were instructed to wear tight shorts/tights, a gym/sports top, T-shirt, or a vest.

**Fig. 1:**
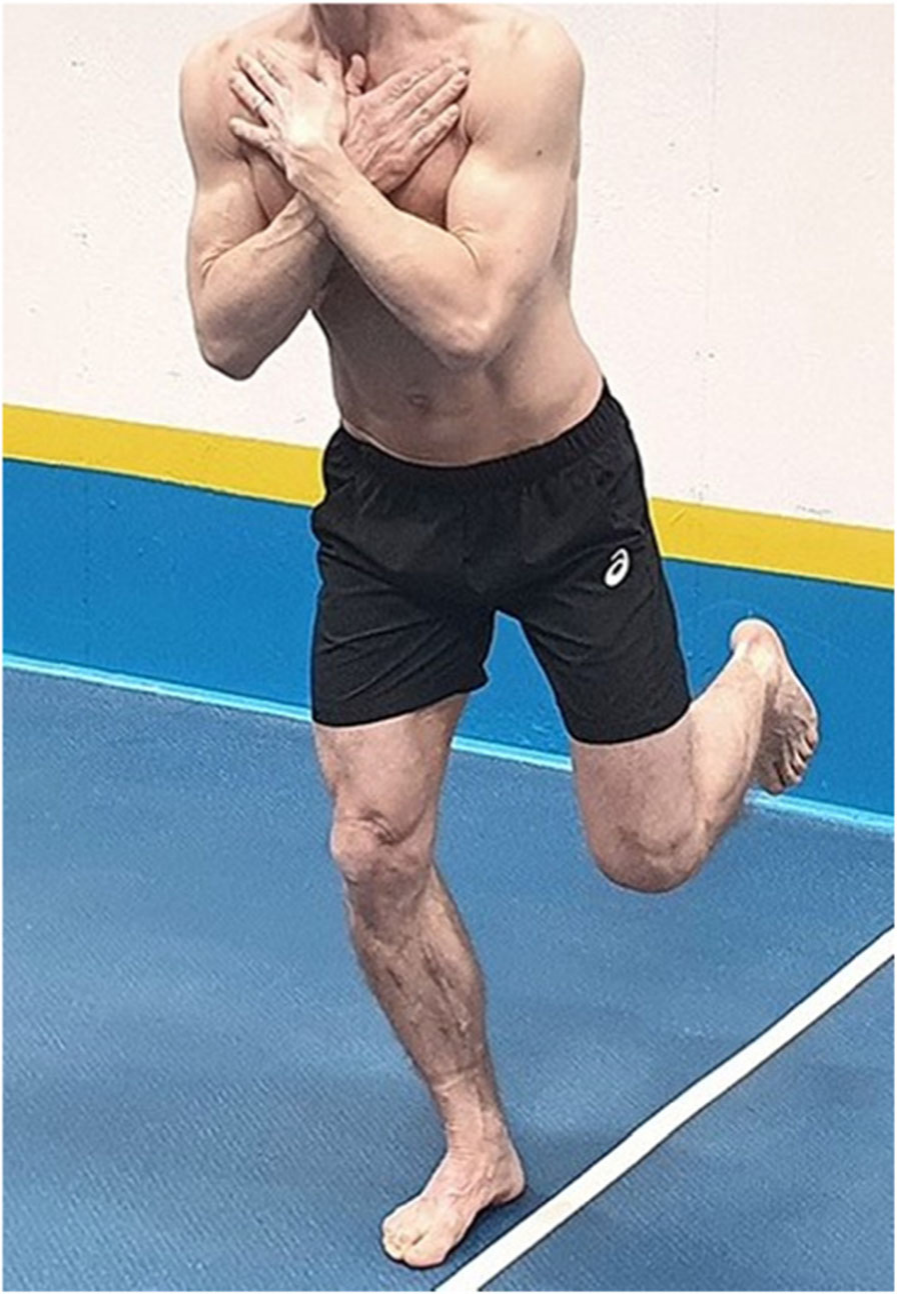
A correct performance of the Single Leg Squat test

The test person was instructed by the pre-recorded video to perform the SLS test with the arms folded across the chest, the non-weight bearing leg flexed so the foot was pointing backwards and the knee pointing straight down to the floor, see Fig. 1. The instruction was to position the weight bearing leg along a sagittal placed sticky tape on the floor, so that the toes pointed straight ahead, and the inside of the foot was parallel to the sticky tape. If the test person could not accomplish this, the foot could be placed in such a way that felt comfortable. The test was performed for both right and left leg and started always with the left leg. The test person was instructed by the pre-recorded video to squat down three times in a controlled manner and with the instruction to go as deep as possible without lifting the heel from the ground or flexing the upper body too much. No additional instructions on how to perform the test was given. All video recordings were scrutinised for quality and “additional ques” such as tattoos, surgical scars or other identifying features which could inflate the reliability, furthermore, no reference standard was available for this material [35].

### Rating procedure

#### Raters

Four physiotherapists were included to assess and rate the video recordings: two experienced and two novices. The experienced raters (1 and 2) had more than 20 years of work experience and the novice pair (3 and 4) had about 4 y. The experienced raters worked at a sports medicine clinic where they used specific movement quality tests at a daily basis [17]. The novice raters had no such previous experience in assessing movement quality and had mostly worked in primary health care.

Ten video recordings of the SLS test, along with written instructions on how to rate and assess the tests, were sent to the raters individually. After one week, one of the authors (JR) held a two-hour educational session with all raters. At this session, the ratings of the 10 video recordings were first discussed to reach a consensus on how to rate the test. This was followed by the individual assessment of 10 additional recordings which were then discussed to achieve a consensus on how to assess the SLS test according to the described criteria. Following the educational session, the four raters received 65 new video recordings of the SLS test to assess individually at their own computers for the study purpose. For intrarater reliability, the raters were sent the same video recording after an adequate wash out period of 10 to 14 days [36]. To minimise bias, the order of the videos in the second assessment was randomised with a web-based research randomiser [37]. On both assessment occasions, the raters were instructed to watch each recording no more than two times without any pausing or slow motion. The use of a ruler or any other tool was not allowed. The raters were in addition blinded to each other, their own ratings, and the test persons demographic such as age, activity level and previous injury.

#### Rating criteria

The rating criteria for the SLS test are described in Table 1. The raters were instructed to observe the video recordings and assess movement deviations from the vertical alignment of the body segments: foot, knee, pelvis, and trunk during the three consecutive squats. The instruction for this multi-segmental approach was to assess the performance of all body segments at the same time and in relation to each other. A deviation of one segment, could only be scored once (one point) even if failed in all of the three squats. No deviation (pass) was scored as 0 points. The total score for the multi-segmental SLS test could range from 0 to a maximum of 4 points. If scored with 0, no deviations were seen in any of the body segments in any of the three squats. If scored with 4 points, deviation (fail) was evident for all four body segments during any of the three squats.

**Table 1:** Rating criteria of the Single Leg Squat test

Observed segments	Correct movement (pass = 0 point)	Movement deviation^**a**^ (fail = 1 point)
**Foot**^**b**^		
The relationship of the sagittal plane and metatarsale 2.	Os metatarsale 2 is in relation to the sagittal plane placed in a lateral angel of ≤10°	The metatarsale 2 is in relation to the sagittal plane placed in a lateral angel that **clearly exceeds 10°**
**Knee**		
Position of the knee in relation to foot.	The centre of the knee is well aligned over the centre of the foot.	The centre of the knee is **clearly** over or medial to digitorum 1.
Medial/lateral perturbation of the knee.	The movement of the knee is vertical and smooth without any medial/lateral shake.	The movement is jerky and **repeated** medial/lateral shake of the knee is seen.
**Pelvis**		
Lateral pelvic shift and/or pelvic rotation.	No lateral pelvic shift and/or pelvic rotation are seen.	The pelvic is **clearly** shifted lateral and/or rotated in any direction.
**Trunk**		
Centre of mass: trunk lean, perturbation and balance.	The trunk is well aligned over the pelvic, hip, knee and foot.	The trunk **clearly** leans in either direction, there is **obvious** trunk sway, loss of balance or movement of the arms.

^**a**^A movement deviation for a segment (1 point) can only be registered one time during the three squats, i.e., a total score of 0–4 points is possible

^**b**^The position of the foot should be observed before the test is executed. If the test person cannot place the foot in the correct position, they are allowed to put the feet where they feel comfortable

**The rater is only allowed to correct the tested person if they:**

1. Flex the upper body as much as the hip, pelvis and groin cannot be observed.

2. If the heel is lifted from the ground and/or if the foot is moved from its starting position.

3. If the test person does not understand the instructions and performs a pistol squat instead of the SLS.

### Statistical analysis

Intra- and interrater reliability was calculated according to Cohen’s kappa statistics together with percentage agreement (PA) and a 95% confidence interval (95% CI) for each separate segment: foot, knee, pelvic and trunk variable [38, 39]. Furthermore, for both intra- and interrater reliability a merged kappa coefficient was calculated for each segment together and denoted as the variable “all segments.” For interrater reliability where multiple raters were compared, a generalised kappa coefficient presented by Fleiss was used [40, 41].

As the magnitude, and interpretation, of the kappa coefficient can be influenced by factors such as prevalence and bias, both prevalence index (PI) and bias index (BI) were calculated and presented together with the kappa statistics (see Tables 3 and 4 for a mathematical clarification) [39]. The effect that prevalence and bias have on the kappa statistics derives from two paradoxes. The first paradox implies that there will be a prevalence effect when there is a predominance of either positive or negative ratings which could be expressed by the PI. A large PI will present a lower kappa and a small PI will present a higher kappa. The effect of PI on kappa is greater for larger values than smaller values [39, 43]. The second paradox relates to the extent of disagreement by the raters on the proportion of positive or negative findings and could be expressed by the BI. A large BI presents a higher kappa, and a small BI presents a lower kappa. The effect of bias is greater when kappa is small and vice versa [39, 43].

As a further support in the interpretation of kappa, the maximum value of kappa (kappa_max_), that could be obtained for the set of data concerned, was also calculated. It is calculated so that the proportions of positive and negative judgements by each rater (i.e., the marginal totals) are taken as fixed, and the distribution of paired ratings (i.e., the cell frequency in the 2 × 2 tables denoted commonly as a, b, c and d) is adjusted to represent the greatest possible agreement. This means that the maximum possible agreement for either presence or absence of the disease will be the smallest of the marginal totals in each case [39]. Kappa_max_ serves to estimate the strength of the agreement while maintaining the proportions of positive ratings demonstrated by each rater. It provides a reference value for kappa that maintain the individual raters overall tendency to assess a condition or select a rating within the constraints obliged by the marginal totals [39]. Finally, the kappa statistics were adjusted for low/high bias and prevalence by calculation of the prevalence-adjusted bias-adjusted kappa (PABAK) [39, 43, 44].

The kappa statistics were interpreted according to Landis and Koch classification of strength of agreement [45]; κ:< 0.00 = poor; κ: 0.00–0.20 = slight; κ: 0.21–0.40 = fair; κ: 0.41–0.60 = moderate; κ: 0.61–0.80 = substantial and κ: 0.81–1.0 = almost perfect. Statistical analysis was performed using STATA version 15.1 with the extension of the “kappaetc” command which handles all kappa presented [42], kappa_max_ was calculated via the web calculator [46]. Furthermore, Microsoft Office Excel version 16 for Windows 10 was used for the calculation of PI and BI.

## Results

Due to poor video quality, three of the 37 included subjects were excluded and further three subjects could only be assessed for one leg. Hence, in total 65 video recordings and 34 test persons (24 women, 10 men) were included in the study. The test persons had a mean (±SD) age of 34 (12) years and about 80% of those were physically active two days or more per week. The test persons characteristics, pain, and activity levels are described in Table 2. All data from the inter- and intrarater reliability assessment of the SLS test are presented in Tables 3 and 4.

**Table 2:** Test subjects’ characteristics, pain, and activity

	All (***n*** = 34)	Women (***n*** = 24)	Men (***n*** = 10)
Age, year			
Mean (SD)	35 (12)	35 (12)	35 (11)
Height, cm			
Mean (SD)	173 (7)	170 (5)	181 (5)
Weight, kg			
Mean (SD)	72 (13)	66 (7)	86 (14)
Physical active ≥2 days/week*			
% of group (*n*)	79% (27)	83% (20)	70% (7)
Pain in regions other than the lower limb			
% of group (*n*)	27% (9)	25% (6)	30% (3)

*Most common physical activities: running/jogging and weightlifting, but yoga, swimming, power walks and cycling were also reported

**Table 3:** Interrater reliability for experienced raters with > 20 years of clinical experience and novice rater with ≤4 years of clinical experience

Raters	PA^**a**^	Kappa^**b**^ (CI 95%)	Kappa_**max**_^**c**^	PI^**d**^	BI^**e**^	PABAK^**f**^ (CI 95%)
**Experienced**						
Rater 1 vs. Rater 2						
Foot	1.0	1.00 (1.00–1.00)	1.0	0.91	0	1.00 (1.00–1.00)
Knee	0.71	0.42 (0.21–0.64)	0.73	0.09	−0.14	0.42 (0.19–0.64)
Pelvis	0.77	0.44 (0.22–0.66)	0.52	0.46	− 0.20	0.54 (0.33–0.75)
Trunk	0.86	0.63 (0.40–0.85)	0.71	0.52	−0.11	0.72 (0.55–0.90)
All segments^g^	0.84	0.57 (0.46–0.68)	0.71	0.50	−0.11	0.67 (0.58–0.76)
**Novice**						
Rater 3 vs. Rater 4						
Foot	0.99	0.66 (0.02–1.00)	0.66	0.95	0.02	0.97 (0.91–1.00)
Knee	0.88	0.41 (0.10–0.72)	0.88	0.69	−0.03	0.69 (0.51–0.87)
Pelvis	0.88	0.44 (0.12–0.76)	0.58	0.75	0.09	0.75 (0.60–0.92)
Trunk	0.89	0.68 (0.46–0.90)	0.68	0.58	0.11	0.79 (0.63–0.94)
All segments^g^	0.90	0.55 (0.40–0.70)	0.79	0.75	0.05	0.80 (0.73–0.87)
**All raters**	**PA**^**a**^	**Generalised kappa**^**h**^ **(CI 95%)**				**PABAK**^**f**^ **(CI 95%)**
Rater 1–4						
All segments^g^	0.85	0.52 (0.43–0.61)				0.70 (0.65–0.76)

^**a**^***PA*** Percent agreement

^**b**^**Kappa:** Cohen’s kappa, calculated by; $\upkappa =\frac{P_o-{P}_c}{1-{P}_c}$

Where; P_o_ (observed agreement) $=\frac{a+d}{n}$ and P_c_ (chance agreement) $=\frac{\left(\frac{f_1x{g}_1}{n}\right)+\left(\frac{f_2x{g}_2}{n}\right)}{n}$

^**c**^**Kappa**_**max**_**:** Is calculated so that the proportions of positive and negative judgements by each rater (i.e. the marginal totals) are taken as fixed, and the distribution of paired ratings (i.e. the cell frequency a,b,c and d) is adjusted so as to represent the greatest possible agreement. That will say, the maximum possible agreement for either presence or absence of the disease is the smaller of the marginal totals in each case [39]

^**d**^***PI*:** Prevalence index, calculated by; $PI=\frac{a-d}{n}$

^**e**^***BI*** Bias index, calculated by; $BI=\frac{b-c}{n}$

^**f**^***PABAK*:** Prevalence-adjusted bias-adjusted kappa, calculated by; *PABAK* = 2*P*_0_ − 1

^**g**^**All segments:** Denotes a merged kappa coefficient for the interrater reliability of each of the segments together (foot, knee, pelvis and trunk)

^**h**^**Generalised kappa:** A generalisation of Scott’s pi presented by Fleiss in order to calculate the interrater reliability of multiple raters [40, 42]

**Table 4:** Intratater reliability for experienced raters with > 20 years of clinical experience and novice rater with ≤4 years of clinical experience

Raters	PA^**a**^	Kappa^**b**^ (CI 95%)	Kappa_**max**_^**c**^	PI^**d**^	BI^**e**^	PABAK^**f**^ (CI 95%)
**Experienced**						
Rater 1						
Foot	1.0	1.0 (1.00–1.00)	1.0	0.91	0.00	1.00 (1.00–1.00)
Knee	0.99	0.97 (0.91–1.00)	0.97	−0.06	0.02	0.97 (0.91–1.00)
Pelvis	0.94	0.86 (0.73–1.00)	0.86	0.32	−0.06	0.88 (0.76–1.00)
Trunk	0.95	0.89 (0.77–1.00)	0.96	0.40	0.02	0.91 (0.80–1.00)
All segments^g^	0.97	0.93 (0.88–0.98)	0.98	0.39	−0.01	0.94 (0.90–0.98)
**Experienced**						
Rater 2						
Foot	1.0	1.0 (1.00–1.00)	1.0	0.91	0.00	1.00 (1.00–1.00)
Knee	0.86	0.71 (0.52–0.89)	0.97	0.25	−0.02	0.72 (0.55–0.90)
Pelvis	0.92	0.74 (0.51–0.96)	0.95	0.65	0.02	0.85 (0.71–0.98)
Trunk	0.99	0.95 (0.85–1.00)	0.95	0.62	0.02	0.97 (0.91–1.00)
All segments^g^	0.94	0.82 (0.73–0.91)	0.99	0.60	0.00	0.89 (0.83–0.94)
**Novice**						
Rater 3						
Foot	0.99	0.66 (0.02–1.00)	0.66	0.95	0.02	0.97 (0.91–1.00)
Knee	0.92	0.72 (0.48–0.96)	0.94	0.68	−0.02	0.85 (0.71–0.98)
Pelvis	0.89	0.17 (−0.22–0.55)	0.88	0.86	−0.02	0.79 (0.63–0.94)
Trunk	0.92	0.69 (0.43–0.95)	0.94	0.71	−0.02	0.85 (0.71–0.98)
All segments^g^	0.93	0.62 (0.45–0.78)	0.96	0.80	0.01	0.86 (0.80–0.92)
**Novice**						
Rater 4						
Foot	0.97	0.48 (−0.16–1.00)	1.0	0.94	0.00	0.94 (0.85–1.00)
Knee	0.91	0.70 (0.47–0.92)	0.70	0.63	0.09	0.82 (0.67–0.96)
Pelvis	0.91	0.69 (0.46–0.93)	0.90	0.63	0.03	0.82 (0.67–0.96)
Trunk	0.92	0.82 (0.66–0.97)	0.82	0.40	0.08	0.85 (0.71–0.98)
All segments^g^	0.93	0.75 (0.64–0.86)	0.83	0.65	0.05	0.85 (0.79–0.92)
**Rater 1–4**	**PA**^**a**^	**Overall kappa**^**h**^ **(CI 95%)**	**Kappa**_**max**_^**c**^	**PI**^**d**^	**BI**^**e**^	**PABAK**^**f**^ **(CI 95%)**
All segments^g^	0.94	0.82 (0.77–0.86)	0.97	0.61	0.01	0.89 (0.86–0.91)

^**a**^**PA:** Percent agreement

^**b**^**Kappa:** Cohens´ kappa, calculated by; $\upkappa =\frac{P_o-{P}_c}{1-{P}_c}$

Where; P_o_ (observed agreement)$=\frac{a+d}{n}$ and P_c_ (chance agreement) $=\frac{\left(\frac{f_1x{g}_1}{n}\right)+\left(\frac{f_2x{g}_2}{n}\right)}{n}$

^**c**^**Kappa**_**max**_**:** Is calculated so that the proportions of positive and negative judgements by each rater (i.e. the marginal totals) are taken as fixed, and the distribution of paired ratings (i.e. the cell frequency a,b,c and d) is adjusted so as to represent the greatest possible agreement. That will say, the maximum possible agreement for either presence or absence of the disease is the smaller of the marginal totals in each case [39]

^**d**^***PI*:** Prevalence index, calculated by; $PI=\frac{a-d}{n}$

^**e**^***BI*:** Bias index, calculated by; $BI=\frac{b-c}{n}$

^**f**^**PABAK:** Prevalence-adjusted bias-adjusted kappa, calculated by; *PABAK* = 2*P*_0_ − 1

^**g**^**All segments:** Denotes a merged kappa coefficient for the intrarater reliability of each segments together (foot, knee, pelvis and trunk)

^**h**^**Overall kappa:** Presents an overall average kappa for the variable all segments for all raters comparing test occasion one and two. Calculated with Cohens’kappa

### Interrater reliability

For the experienced raters (rater 1 vs. 2), the interrater reliability varied between a “moderate” agreement for the knee variable (ĸ = 0.42, 95% CI 0.21–0.64) and “almost perfect” for the foot (ĸ = 1.00, 95% CI 1.00–1.00). The pelvic variable reached a “moderate” agreement (ĸ = 0.44, 95% CI 0.22–0.66) and the trunk variable a “substantial” agreement (ĸ = 0.63, 95% CI 0.40–0.85). For the variable all segments, a “moderate” agreement (ĸ = 0.57, 95% CI 0.46–0.68) was obtained. The largest difference between the calculation of kappa and kappa_max_ was seen for the knee variable (ĸ = 0.42 vs. kappa_max_ = 0.73), no greater difference was seen between kappa and PABAK.

For the novice raters (rater 2 vs. 3), the interrater reliability varied between a “moderate” agreement for the knee variable (ĸ = 0.41, 95% CI 0.10–0.72) and “substantial” for the trunk (ĸ = 0.68, CI 95% 0.46–0.90). The pelvic variable reached a “moderate” agreement (ĸ = 0.44, 95% CI 0.12–0.76) and the foot variable a “substantial” agreement (ĸ = 0.66, 95% CI 0.02–1.00). For the variable all segments, a “moderate” agreement (ĸ = 0.55, 95% CI 0.40–0.70) was obtained. The largest difference between the calculation of kappa and kappa_max_ was seen for the knee variable (ĸ = 0.41 vs. kappa_max_ = 0.88). In general, PABAK was slightly higher than the kappa coefficient.

For all raters together (rater 1–4), the variable all segments obtained a generalised kappa coefficient of “moderate” agreement 0.52 (95% CI 0.43–0.61), while PABAK reached “substantial” agreement (0.70, 95% CI 0.65–0.76).

### Intrarater reliability

For the experienced raters, the intrarater reliability varied between “substantial” (knee variable; ĸ = 0.71, 95% CI 0.52–0.89) to “almost perfect” agreement (foot variable; ĸ = 1.00, 95% CI 1.00–1.00). The pelvic variable reached a “substantial” agreement for rater 2 (ĸ = 0.74, 95% CI 0.51–0.96) and an “almost perfect” agreement for rater 1 (ĸ = 0.86, 95% CI 0.73–1.00), the trunk variable reached “almost perfect” agreement for both experienced raters (rater 1: ĸ = 0.89, 95% CI 0.77–1.00; rater 2: ĸ = 0.95, 95% CI 0.85–1.00). For the variable all segments an “almost perfect” agreement was obtained for both raters (rater 1: ĸ = 0.93, 95% CI 0.88–0.98; rater 2: ĸ = 0.82, CI 95% 0.73–0.9). The largest difference between the calculation of kappa and kappa_max_ was seen for rater 2 and the variables knee (ĸ = 0.71 vs. kappa_max_ = 0.97) and pelvic (ĸ = 0.74 vs. kappa_max_ = 0.95). No greater difference was seen between kappa and PABAK.

For the novice raters the intrarater reliability ranged from “slight” agreement (pelvic variable; ĸ = 0.17, 95% CI -0.22-0.55) to “almost perfect” (trunk variable; ĸ = 0.82, 95% CI 0.66–0.97).

The foot variable varied between a “moderate” agreement for rater 4 (ĸ = 0.48, 95% CI -0.16-1.00) and a “substantial” agreement for rater 3 (ĸ = 0.66, 95% CI 0.02–1.00), the knee variable reached “substantial” for both novice raters (rater3: ĸ = 0.72, 95% CI 0.48–0.96; rater 4: ĸ = 0.70, 95% CI 0.47–0.92) and the variable all segments reached “substantial” agreement for both raters (rater 3: ĸ = 0.62, 95% CI 0.45–0.78; rater 4: ĸ = 0.75, 95% CI 0.64–0.86). The largest difference between the calculation of kappa and kappa_max_ was seen for rater 3 and the variable pelvic (ĸ = 0.17 vs. kappa_max_ = 0.88) and for rater 4 and the variable foot (ĸ = 0.48 vs. kappa_max_ = 1.0). These segments also showed a great difference between kappa and PABAK; pelvic (ĸ =0.17, 95% CI − 0.22-0.55 vs. PABAK = 0.79, 95% CI 0.63–0.94) and foot (ĸ =0.48, 95% CI − 0.16-1.00 vs. PABAK = 0.94, 95% CI 0.85–1.00).

For the variable all segments, an overall average kappa was calculated for all raters (rater 1–4) which reached “almost perfect” agreement (ĸ = 0.82, 95% CI 0.77–0.86), no greater difference was seen between kappa and PABAK.

## Discussion

The aim of the present study was to investigate the intra- and interrater reliability of a standardised multi-segmental SLS test. All in all, the SLS test showed an acceptable intrarater reliability for all raters and all separate variables (foot-, knee-, pelvis- and trunk). For all variables, the agreement was classified as “moderate” or better than so (ĸ ≥0.41), except for the pelvic variable for one of the novices raters. Regardless of the raters experience, and for the variable all segments, the SLS test demonstrated a “moderate” interrater reliability and an “almost perfect” intratater reliability.

In general, reliability is considered to depend on several factors, such as the complexity of the rating scale (dichotomised or multiple-rating, number of segments assessed), the definitions of the rating criteria, the velocity of the tests and the examiner’s training and clinical experience [33, 47]. Compared to our findings a recent meta-analysis on the intra- and interrater reliability of different SLS tests (SLS, FSD and LSD) [20] included 17 studies investigating the reliability of multi-segmental SLS tests. Seven of those reported higher reliability [7, 23, 24, 48–51], and 10 equivalent reliability [17–19, 22, 52–57] compared to our results. The reason for the higher reliability might be due to several factors, including the methodological setup and actual test performance. Our study used a convenient sample of 34 persons, and 65 video recordings, without any categorisation and equal distribution of the performed tests on the video recordings (i.e., good, fair, or poor performance). In addition, our raters were instructed to watch the video recordings only twice without any pausing or slow-motion. Crossly et al. [7] and Herman et al. [56] presented “moderate” to “substantial” interrater reliability but used in contrast to our study a consensus panel and six to 15 video recordings, that unlike the other recordings, had been rated with a 100% agreement by the panel at their first rating. Furthermore, McKeown et al. [18] who presented “moderate” interrater reliability allowed their raters’ to watch 17 video recordings an unlimited number of times, both in real time and in slow motion. The results of these studies show that the methodology of a study is affecting the results of reliability to a large extent. We have in our study aimed to resemble a clinical situation and our intention was to evolve a less complex and well-defined multi-segmental SLS test which would be easily used regardless of the examiner’s education or clinical experience. The complexity was reduced by using a dichotomous rating scale, not including all possible segments in the kinetic chain, and by taking less movement deviations per segment into account. We used individual training of the raters using 10 video clips and in addition a two-hour educational session to improve the ratings. Seven comparable studies which included both experienced and unexperienced physiotherapists, physiotherapy students and novice athletic therapists showed both better and equivalent reliability than our study but used twice as much (or more) education if not taking the individual training of 10 video clips into account [17, 22, 23, 48, 50, 51, 55]. Thus, it seems that the results from the present multi-segmental SLS test, despite less education, is in accordance with other multi-segmental reliability studies on the SLS test.

It could be discussed if some facilitating utilities assisting the assessment may lead to better reliability. Three comparable studies which showed “substantial” reliability used markers on the floor to indicate the first or second toe, and in addition markers on the tuberosities tibia [23, 50, 55]. It is not really possible to say if their interrater reliability was due to those markers as they also used an extensive education program (4-, 5- and 20-h respectively) and a different methodological setup in comparison to the present study. However, it is interesting to note that Rabin et al. [51, 55] who performed two almost identical studies, except for the population and facilitating utilities, reached “moderate” reliability in the first study [55] and “almost perfect” in the second study [51]. In their second study, they used a vertical pole in addition to the markers, positioned in front of the tested subjects to enhance the visibility of the movements of the lower limb. On the other hand, it might be more likely that the use of the same raters with an additional four-hour education would have made a greater impact on the reliability than the utilities. Our study used a sticky tape placed on the floor with the purpose to mark the sagittal plane when assessing the habitual placement of the foot. It could be so, that the sticky tape facilitated the assessment of the foot but not the knee, which might be reflected by the constant relatively lower kappa statistics for the knee variable.

To our knowledge, so far, no study has investigated the intra- and interrater reliability of the foot position in relation to the sagittal plane. More commonly, the pronation of the foot is considered as a movement deviation and therefore included in the assessment of a SLS test. To provide for the position of the foot, some studies used a sticky tape shaped as a T, or just a verbal instruction to align the foot in the sagittal plane [21, 53, 54, 58], but far from all studies report a standardised foot position. Our study used a standardised foot position which has been described as an alignment of the second metatarsal in relation to the sagittal plane (a lateral angel of ≤10°) [59]. The position of the foot is important as it acts as a specific reference point in the assessment of the knee, but also as an overall reference for the whole kinetic chain. If a test person shows a habitual foot position with a lateral angle ≥10°; it is the authors opinion that the knee in most of these cases will be assessed as a failure. This due to the knee will be positioned medial to the foot or greater toe from the start, even though the movement of the knee might be smooth, vertical, and sagittal aligned. This could also apply to the whole kinetic chain, which could be well aligned over a lateral rotated foot. On the other hand, to force someone into a smaller lateral angel than their habitual foot position might produce movement deviations further up in the chain. This discussion is lacking in the literature and further studies are warranted to investigate the relationship of the foot position and the outcome of a multi-segmental SLS test.

The present study used recorded video clips to observe and assess the performed SLS test. Video recordings were chosen to standardise the testing procedure enabling several raters to assess identical test performances. However, in a clinical situation the therapist most likely will observe and assess the SLS test live meaning that the present method used lowers the tests’ ecological validity. As for any test, it is important that the patient understands the instructions of how to perform the test. We therefore recommend that the instructions to perform the present standardised SLS test (Additional file 2) are followed. To assess a SLS test using a multi-segmental approach, all segments are assessed at the same time and in relation to each other. This means that the rater needs to assess the whole kinetic chain at the same time and not one segment at a time. This way of assessing the SLS test has previously been described in studies of the SLS test [6, 7, 17]. In addition, we do not propose a composite score for the SLS test [24, 55] since a total score conceals the information on which segment or segments that have been scored as fail.

### Methodological considerations

Three major strengths of the present study are the use of different statistical computations, the methodological standardisation based on the Quality Appraisal for Reliability Studies checklist (QAREL) [35, 60], and that the proposed SLS test was based on findings from previous studies investigating the SLS tests measurement properties [20, 32, 33].

As the magnitude of kappa is influenced by different factors, for example prevalence and bias, a comparison of the strength of kappa across studies with different statistics could be difficult [39, 61]. In this context, kappa_max_ and PABAK acts as a further support in the judgement of the magnitude of an obtained kappa coefficient [39] and enables a robust result in present study. Hence, when taking the prevalence and bias effects acting on the kappa coefficient of the present study in account and considering the particular methodological context in which the study is conducted, we conclude that the proposed multi-segmental SLS test is reliable enough to be used on an active population in the clinical practice. For reliability and validity studies a sample size of at least 50 measures is recommended [61, 62]. The present study used 260 separate measures for each rater (65 video recordings and 4 segments), which could be considered as an appropriate amount of data fulfilling the requirement of at least 50 data points. Even though 3D and 2D studies report joint kinematics with fair to good agreement over time, the SLS, FSD and LSD joint kinematics have not yet been adequately assessed for within-subject reliability using visual assessment [20, 33]. The use of video recordings in present study could therefore be considered a strength for the assessment of the intrarater reliability, since the recordings eliminate the normal within-subject variety. On the other hand, a drawback with video recordings is that the authentical patient-clinician interaction is lost. The study population was a convenience sample of both men (29%) and women (71%) with an average age of 34 (±SD 12) years who were relatively active, mostly with running/jogging and weightlifting. This is an appropriate subgroup of subjects where the SLS test could be applicated, increasing the external validity. However, no further generalisations to another population can be made from our findings, and a more equal distribution of men and women would have been preferable. Another limitation of the present study is that no further generalisation across raters or clinicians can be done from our four raters. In contrast to this, Herman et al. [56] included 142 physiotherapists with varying experience and reached equal reliability as present study. On the other hand, as mentioned above, Herman et al. [56] used a methodological setup which might not be comparable with present study. Also, Teyhen et al. [50] used a multi-rater setup, and included 29 doctoral students with less clinical experience, they used an extensive 20-h education program and reach slightly better reliability than present study.

## Conclusion

We propose a SLS test, analysed in a study with a rigorously methodological set up, taking the functional aspects of sport-related actions into account, and considering the whole kinetic chain. Regardless of the raters’ experience and with a common two-hour education, the present study shows a “moderate” interrater reliability and an “almost perfect” intrarater reliability for the variable all segments. Thus, we conclude that the standardised multi-segmental SLS test is reliable enough to be used in an active population.

## Supplementary Information

**Additional file 1:.** Quality Appraisal for Reliability Studies checklist (QAREL). Contains a table with the 11 items of Quality Appraisal for Reliability Studies checklist (QAREL), their answers and explanations.

**Additional file 2:.** Instructions to the performance of the Single Leg Squat test. Contains written instructions to the test leader regarding the test performance and verbal instructions to the tested subjects.

## Data Availability

The datasets generated and/or analysed during the current study are not publicly available due to ethical regulation at the Karolinska Institute but are available from the corresponding author on reasonable request.

## Abbreviations

3D: 3-dimensional
2D: 2-dimensional
95% CI: 95% confidence interval
BI: Bias index
FSD: Forward Step Down
LSD: Lateral Step Down
PA: Percent agreement
PABAK: Prevalence-adjusted bias-adjusted kappa
PI: Prevalence index
QAREL: Quality Appraisal for Reliability Studies checklist
SLS: Single Leg Squat
