# Supplementary material for: Visual assessment of movement quality: a study on intra- and interrater reliability of a multi-segmental single leg squat test
Source: BMC Sports Sci Med Rehabil. 2021 Jun 8;13:66. doi: 10.1186/s13102-021-00289-x (PMC8186063; doi:10.1186/s13102-021-00289-x)
Supplement: Supplementary file 1 — Additional file 1:. Quality Appraisal for Reliability Studies checklist (QAREL). Contains a table with the 11 items of Quality Appraisal for Reliability Studies checklist (QAREL), their answers and explanations. [file 13102_2021_289_MOESM1_ESM.pdf]

Additional file 1

Quality Appraisal for Reliability Studies checklist (QAREL)

Question	Answer	Explanation
1.<br>Was the test evaluated in a sample of subjects who were representative of those to whom the authors intended the results to be applied?	Yes	Active healthy subjects, 34 (±12) years, is a representative sample of subjects to whom the results are intended to be applied.
2.<br>Was the test performed by raters who were representative of those to whom the authors intended the results to be applied?	Yes	Physiotherapists regardless of experience.
3.<br>Were raters blinded to the findings of other raters during the study?	Yes	
4.<br>Were raters blinded to their own prior findings of the test under evaluation?	Yes	
5.<br>Were raters blinded to the results of the accepted reference standard or the disease status for the target disorder (or variable) being evaluated?	Yes	Raters were blinded to the disease status of the subjects. No accepted reference standard was available for this set of subjects.
6.<br>Were raters blinded to clinical information that was not intended to be provided as part of the testing procedure or study design?	Yes	
7.<br>Were raters blinded to additional cues that were not part of the test?	Yes	Video recordings were screened for additional cues such as scares, tattoos or orthoses that could bias the test findings.
8.<br>Was the order of examination varied?	Yes	Randomized.
9.<br>Was the stability (or theoretical stability) of the variable being measured taken into account when determining the suitability of the time-interval among repeated measures?	Yes	Present study used video recordings which eliminates possible within-subjects variety with testing from time to time such as intrarater reliability.
10.<br>Was the test applied correctly and interpreted appropriately?	Yes	The test is described in detail including rational, procedure and interpretation. The test is based on previous findings from other reliability studies on multi-segmental SLS test.
11.<br>Were appropriate statistical measures of agreement used?	Yes	For categorical data: kappa statistics with confidence interval, percent agreement and prevalence-adjusted bias-adjusted kappa was presented.

The items can be answered: yes, no, unclear, and not applicable.
