# Supplementary material for: Visual assessment of movement quality: a study on intra- and interrater reliability of a multi-segmental single leg squat test
Source: BMC Sports Sci Med Rehabil. 2021 Jun 8;13:66. doi: 10.1186/s13102-021-00289-x (PMC8186063; doi:10.1186/s13102-021-00289-x)
Supplement: Supplementary file 2 — Additional file 2:. Instructions to the performance of the Single Leg Squat test. Contains written instructions to the test leader regarding the test performance and verbal instructions to the tested subjects. [file 13102_2021_289_MOESM2_ESM.pdf]

Instructions to the performance of the Single Leg Squat test

Instructions to the test leader and the tested subjects

- The test is performed in shorts/tights, a sport top, T-shirt, or a vest.
- The test person is instructed to stand with his/her feet on either side of a sticky tape that is placed in the sagittal plane at the floor, the medial arches of the feet should be in line with the tape (or the lateral angle of sagittal plane and the metatarsale 2 should not exceed 10°). If the test person cannot do that, he/she is allowed to put the feet where they feel comfortable.
- The test is always performed with the left leg first.
- The tested person is allowed to practice the test three times before the test situation.
- During the test trial and the actual test, the rater are only allowed to correct the tested person if they:

    1. Flex the upper body as much as the hip, pelvic and groin cannot be observed.
    2. If the heel is lifted from the ground and/or if the foot is moved from its starting position.
    3. If the test person does not understand the instructions and performs a pistol squat instead of a Single Leg Squat.

Verbal instruction before the test

1. Stand on each side of the line with the inside of your feet approximately 5-10 cm from the line.

2. Your toes should point straight ahead in the room and the inside of our foot should be parallel to the line at the floor.

3. Place your hands across your chest.

4. You are soon about to perform three single leg squats in controlled manner and as deep as possible without lifting your heel from the ground. It is important that you do not bend your upper body forward during the movement.

5. Stand on your left leg by bending your right knee so that your right foot is pointing backwards, and your right knee is pointing straight down to the floor.

6. Now, perform three single leg squats.

7. When you are done, return to the starting position on two legs.
